# Diabetic Retinopathy –Incidence And Risk Factors In A Community Setting- A Longitudinal Study

**DOI:** 10.1080/02813432.2018.1487524

**Published:** 2018-06-27

**Authors:** Michal Shani, Tali Eviatar, Doron Komaneshter, Shlomo Vinker

**Affiliations:** a Department of Family Medicine, Clalit Health Services, Central District, Rehovot, Israel;; b Department of Family Medicine, Sackler Faculty of Medicine, Tel Aviv University, Tel Aviv, Israel;; c Chief physician office, Clalit Health Service, Tel Aviv, Israel

**Keywords:** diabetic retinopathy, diabetes mellitus, non-proliferative diabetic retinopathy, proliferative diabetic retinopathy, community care

## Abstract

**Aim:** To evaluate the natural history of diabetic retinopathy (DR) in diabetic patients and to assess long term risk for other chronic diseases associated with DR.

**Methods:** Retrospective, community-based study. Diabetics who underwent their first fundoscopic examination during 2000–2002, and had at least one follow- up examination by the end of 2007 were included. The primary outcome was the development of DR (proliferative diabetic retinopathy (PDR), non PDR (NPDR) or macular edema.

Patients were followed for another 9 years for documentation of new diagnosis of related diseases.

**Results:** 516 patients' (1,032 eyes) records were included and were followed first for an average of 4.15 ± 1.27 years. During follow-up, 28 (2.7%) of the total 1,032 eyes examined were diagnosed with PDR. An additional 194 (18.8%) eyes were diagnosed with new NPDR. The cumulative incidence of NPDR was 310/1,032 (30.0%). All the patients who developed PDR had prior NDPR. By the end of the 9 years extended follow up, patients with NPDR had a greater risk for developing chronic renal failure HR = 1.71 (1.14–2.56), ischemic heart disease HR = 1.57 (1.17–2.09), and had an increased mortality rate HR = 1.26 (1.02–1.57)

**Conclusion:** DR is associated with a higher rate of diabetes complications. Patients with DR should be followed more closely.

**Key points**
- During a mean follow-up of 4.5 years, the cumulative incidence of diabetic retinopathy in a community cohort was 18.8%.
- NDPR (non-proliferative diabetic retinopathy) is a predictor of PDR (proliferative diabetic retinopathy).
- In a real life setting NPDR is a marker of a poorer prognosis.
- Patients with NDPR should be monitored more closely.

## Introduction

Diabetes retinopathy is the leading cause of incident blindness among adults between the ages of 20–74 in the United States [1] and also a leading cause for blindness in Israel [2]. During the first 20 years following diagnosis, nearly all individuals with type 1 diabetes (T1DM), and over 60% of those with type 2 diabetes (T2DM), are likely to develop diabetic retinopathy (DR). At the time of diagnosis, 21% of those with T2DM show signs of diabetic retinopathy [3].

The Wisconsin Epidemiologic Study of Diabetic Retinopathy (WESDR) [4] and the Diabetes Control and Complications Trial (DCCT) [5] demonstrated an association between better glycemic control and reduced risk for retinopathy in individuals with T1DM. Findings of the Early Treatment Diabetic Retinopathy Study (ETDRS) [6], UK Prospective Diabetes Study (UKPDS) [7], and Action to Control Cardiovascular Risk in Diabetes Study (ACCORD) [8], demonstrated a similar association in individuals with T2DM., The ten-year post-trial follow-up of the UKPDS showed that the benefit of reduced glycemia persisted over time. These studies failed to demonstrate an association between retinopathy and ischemic heart disease and cerebrovascular accident in diabetic patients. In these cases, despite an early loss of glycemic differences between groups treated with intensive therapy (sulfonylureas and insulin) and conventional therapy (dietary restriction), the risk for microvascular complications continued to be 24% lower in the former than in the latter [9].

Diabetic retinopathy is categorized as non-proliferative DR (NPDR) and proliferative DR (PDR), according to the clinical and pathological pattern of the disease. Diabetic macular edema (DME) may further complicate each of these types of DR.

NPDR is characterized by retinal blood vessel microaneurysms and retinal hemorrhages, which may lead to hypoperfusion and ischemia of the affected retinal territory. NPDR is usually asymptomatic, though some patients complain of impaired vision acuity or color discrimination. Further ischemic damage to the retina may promote the production of angiogenic factors, which can result in neovascularization of the retinal surface or even the optic disc; this being characteristic of PDR [10]. The fragile new blood vessels are prone to disintegration and cause bleeding at the retinal surface or in the vitreous body, which may further cause traction retinal detachment. These complications may lead to severe and sometimes irreversible visual impairment [10].

Ophthalmic microvascular complications of diabetes can be delayed or even prevented when diagnosed and treated at early stages. Hence, early identification of at-risk patients is of particular importance.

The aim of this study was to evaluate the natural course of diabetic retinopathy in diabetic patients in real life setting and to assess the significance of factors associated with the development of NPDR.

## Methods

This is a retrospective, longitudinal, community-based study. The study was conducted in the Central District of “Clalit Health Service” (CHS) in Israel.

Patients: Individuals with diabetes mellitus who underwent their first fundoscopic examination between the years 2000 and 2002, and had at least one follow- up examination by the end of 2007. Those diagnosed with PDR (in one eye or both eyes) on their first fundus examination were excluded.

Data was retrieved from the computerized medical records of an urban general ophthalmologic clinic. A total of 731 consecutive patient records were reviewed, of which, 215 did not match the inclusion criteria (15 had PDR in the first examination, and 200 did not have a follow up examination until 2007). The primary outcome was the development of diabetic retinopathy (PDR, NPDR) or DME at the follow up ophthalmologist visit. Data attained from patient charts was comprised of demographic variables: age when entered the study, gender, socio-economic level (low economic status was determined according to the National Security Institute of Israel), as well as diabetes treatment at the beginning of follow-up (diet, oral medications, insulin, or a combination of oral medications and insulin), diagnoses of hyperlipidemia, hypertension, and obesity: smoking history, and chronic diseases at baseline, glycemic control according to HbA1c, mean levels during follow up of low density lipoprotein (LDL) cholesterol and of blood pressure.

Eye examinations were performed by ophthalmologists in the community clinics, and included an eye fundus examination using an indirect ophthalmoscopy with a slit-lamp following a pharmacologic midriasis of the inspected eye. Test results were categorized according to the International Clinical Diabetic Retinopathy and Diabetic Macular Edema Disease Severity Scale [11].

We followed this cohort of diabetic patients for another 9 years (until December 31^st^ 2016) and looked for any new diagnosis in patient's file of ischemic heart disease, congestive heart failure, peripheral vascular disease, cerebrovascular accident, leg amputation, chronic renal failure, and all-cause mortality. We used Intra-vitreous injection of bevacizumab or ranibizumab as markers of advanced retinal disease.

Data was statistically analyzed using STATA 8.0 software. The chi-square test was used to calculate categorized variables, and Student's test and ANOVA to calculate continuous variables. Multivariate analysis was performed using a logistic regression model. Cox analysis was performed to assess time till new diagnosis of the chronic diseases tested.

## Results

A total of 516 patients (1,032 eyes) records met inclusion criteria and patients were followed in the first phase for an average of 4.15 ± 1.27 years. During the first follow-up period, 32 patients (6.2%) died. Of the 484 patients who were alive at the end of 2007, 240/484 (49.6%) died during the second follow up period. Demographic data of the study population at baseline is presented in Table 1.

**Table 1.** Demographic and clinical characteristics of 516 patients at baseline.

Characteristics	N = 516 patients
Gender (% males)	47.7%
Age (mean ± SD, range)	10.8 ± 64.9 (16-87)
Low socio-economic status (%)	26.6%
HbA1c (gr%) (mean ± SD, range)	1.3 ± 7.5 (4.8-13)
DM treatment at baseline	
Diet only	28.5%
Oral medications	56.4%
Insulin (with or without oral hypoglycemic)	15.1%
Cardio-Vascular Risk Factors	
LDL cholesterol mg/dL (mean ± SD, range)	23.3 ± 110.4 (48-211.8)
systolic BP mmHg (mean ± SD, range)	14.3 ± 138.8 (100-205)
diastolic BP mmHg (mean ± SD, range)	5.9 ± 78.0 (52-98.5)
Creatinine (gr/dL) (mean ± SD, range)	0.4 ± 1.04 (0.6-5.7)
BMI >30	34.1%
Smoking	24.8%
DM complications and cardiovascular diseases	
Ischemic heart disease	42.1%
Congestive heart failure	19.0%
Microalbuminuria	6.2%
Chronic Renal Failure	15.9%
Peripheral Vascular Disease	16.9%
Cerebro-Vascular accident	16.5%
Carotid artery stenosis	9.5%

116 (11.2%) eyes were diagnosed with NPDR at baseline. During follow-up, 28 (2.7%) of the total 1,032 eyes examined were diagnosed with PDR. An additional 194 (18.8%) eyes were diagnosed with new NPDR during follow-up. The cumulative incidence of NPDR was 310/1,032 (30.0%).

DME was documented in 14 (1.4%) eyes at the first ophthalmologic examination, and in 43(4.2%) during the first follow-up period. The cumulative incidence of DME was 5.6%. DME was detected in 11/28 (39.3%) of the eyes diagnosed with PDR and in 46/1,004 (4.6%) eyes without PDR (*p* < 0.0001).

The characteristics of the patients who developed NPDR during first follow up period are described in table II.

**Table 2.** Characteristics of eyes who developed NPDR during the first study period vs. eyes without retinopathy at baseline*.

	No retinopathy (N = 721)	New NPDR during study period (N = 194)	p-value
Gender (male)	46.3%	49.5%	NS
Age	64.7 ± 11.6	66.4 ± 8.5	NS
Low SES	24.5%	26.8%	NS
Diet only	37%	12.4%	<0.001
Oral hypoglycemic	55.3%	61.3%	
Insulin use	7.6%	26.3%	<0.001
HbA1c at beginning of study	7.6 ± 1.5	8.6 ± 1.8	<0.001
Average HbA1c during study period	7.3 ± 1.2	7.9 ± 1.3	<0.001
LDL cholesterol	112.4 ± 23.8	104.1 ± 22.9	<0.001
Total protein secretion in urine	26.5%	41.8%	<0.001
Microalbuminuria	22.3%	34.0%	
Overt proteinuria	4.2%	7.7%	
Creatinine	1.0 ± 0.3	1.0 ± 0.4	NS
Systolic BP	137.9 ± 13.8	140.0 ± 15.2	NS
Diastolic BP	78.3 ± 5.4	77.7 ± 6.6	NS
HTN	77.4%	84.0%	0.05
BMI >30	31.9%	40.7%	0.05
Smoking	22.6%	32.5%	<0.01
Ischemic heart disease	37.9%	53.6%	<0.001
Congestive heart failure	16.1%	25.8%	<0.01
Peripheral vascular disease	13.7%	26.8%	<0.001
s/p CVA	14.7%	18.0%	NS

* Out of 915 eyes without retinopathy at study beginning.

All of the patients who developed PDR had prior NPDR. A multivariate regression model (data not shown) showed treatment with insulin to be the most predictive parameter associated with the development of NPDR (OR 3.5, *P* < 0.001).

By the end of the 9 years extended follow up, patients with NPDR were at a greater risk for developing chronic renal failure, ischemic heart disease and heart failure. They had a significantly higher mortality rate (table III).

**Table 3.** Percentage of eyes of diabetic patients who developed new diagnosis during extended follow-up 2008-2016 (and didn’t have the diagnosis before the end of 2007), and the hazard ratio to develop the new diagnosis.

	NPDR-	NPDR +	HR (CI)	Adjusted HR (CI)
Intravitrous treatment with bevacizumab or ranibizumab	(5.0%) 34/685	9.9% (28/283)	2.18 (1.37-3.46)	2.54 (1.57-4.10)
IHD	40.3% (268/665)	51.9% (150/289)	1.40 (1.06-1.84)	1.57 (1.17-2.09)
CHF	16.6% (105/630)	26.7% (71/266)	1.66 (1.15-2.39)	1.51 (1.02-2.21)
CVA	20.6% (128/620)	21.4% (58/270)	0.18 (0.02-1.40)	0.24 (0.03-1.83)
Leg amputation	0.8% (8/713)	1.3% (4/303)	1.64 (0.46-5.8)	1.50 (0.37-6.1)
PVD	19.6% (133/680)	17.6% (49/278)	0.52 (0.23-2.11)	0.53 (0.17-1.64)
CRF	18.2% (108/592)	29.0% (72/248)	1.86 (1.27-2.73)	1.71 (1.14-2.56)
Death	43.4% (344/793)	41.5% (73/176)	0.98 (0.80-1.22)	1.26 (1.02-1.57)

## Discussion

During a mean follow-up of 4.5 years in phase one, the cumulative incidence of diabetic retinopathy in a community cohort in real life setting was 18.8%. These findings are compatible with other studies. In the UKDPS 22% of patients developed DR within 6 years [12]. In another community-based study retinopathy rate was 28% after 9 years. [13] DME was detected in 39.3% of eyes with PDR.

In the current study, better glycemic control, was associated with reduced risk of retinopathy, supporting data from other studies [5,7] Insulin treatment was associated with NPDR development, regardless of the HbA1c level. It may be a proxy for disease length or poorer glycemic control. Age, gender and socioeconomic status were not related to development of NPDR for patients who were seen by an ophthalmologist. It is important to bear in mind that disparities in diabetes care may exist even in countries where access to care is free [14].

Our findings support an association between retinopathy and other late diabetes complications. The positive association between NPDR and protein secretion and chronic renal failure support the well-established association between retinopathy and protein excretion [15]. The association between retinopathy and ischemic heart disease was not consistent over various studies. Although the Framingham study reported an association between diabetic retinopathy and cardiovascular disease, including ischemic heart disease many years ago [16] other studies did not. In a meta-analysis the risk ratio for ischemic heart disease in type 2 diabetes was 1.81 which is compatible with our findings [17]. Higher risk for heart failure was reported before [18]. Higher rate of ischemic heart disease may explain the increased risk for congestive heart failure. Higher mortality rate was also observed before [18] as retinopathy may be a marker of advanced disease.

Study limitations: The main limitation of the study is the determination of retinopathy by fundus examination and not by photographs. Nevertheless all examinations were performed by an ophthalmologist after pupil dilation and using an indirect ophthalmoscope slit-lamp

The overall long follow up period (an average follow up of 13.5 years) revealed that in real life setting NPDR is a marker of poorer prognosis, higher risk of cardiovascular diseases and chronic renal failure, and higher mortality rates.

Screening for retinopathy may add important information to the primary care physician about his/her diabetic patient. Our findings may also help to differentiate between patients according to their risk for retinopathy. Patients with no signs of retinopathy may enjoy longer intervals between ophthalmologist visits as was offered forT1DM patients [19].

In conclusion diabetic retinopathy is prevalent and associated with other long term diabetic complications as it is a marker of an advanced disease. NDPR is a predictor of PDR. Patients with NDPR, should be monitored more closely both by an ophthalmologist and by their family physician.
